# Probiotics *Streptococcus salivarius* 24SMB and *Streptococcus oralis* 89a interfere with biofilm formation of pathogens of the upper respiratory tract

**DOI:** 10.1186/s12879-018-3576-9

**Published:** 2018-12-13

**Authors:** Alessandro Bidossi, Roberta De Grandi, Marco Toscano, Marta Bottagisio, Elena De Vecchi, Matteo Gelardi, Lorenzo Drago

**Affiliations:** 1 Laboratory of Clinical Chemistry and Microbiology, IRCCS Orthopedic Institute Galeazzi, Milan, Italy; 2 Department of Biomedical Sciences for Health, Laboratory of Clinical Microbiology, University of Milan, Milan, Italy; 3 Department of Basic Medical Science, Otolaryngology Unit, Neuroscience and Sensory Organs, University of Bari Aldo Moro, Bari, Italy

**Keywords:** Biofilm, *Streptococcus oralis*, *Streptococcus salivarius*, Probiotics, Respiratory tract infections

## Abstract

**Background:**

Infections of the ears, paranasal sinuses, nose and throat are very common and represent a serious issue for the healthcare system. Bacterial biofilms have been linked to upper respiratory tract infections and antibiotic resistance, raising serious concerns regarding the therapeutic management of such infections. In this context, novel strategies able to fight biofilms may be therapeutically beneficial and offer a valid alternative to conventional antimicrobials. Biofilms consist of mixed microbial communities, which interact with other species in the surroundings and communicate through signaling molecules. These interactions may result in antagonistic effects, which can be exploited in the fight against infections in a sort of “bacteria therapy”. *Streptococcus salivarius* and *Streptococcus oralis* are α-hemolytic streptococci isolated from the human pharynx of healthy individuals. Several studies on otitis-prone children demonstrated that their intranasal administration is safe and well tolerated and is able to reduce the risk of acute otitis media. The aim of this research is to assess *S. salivarius* 24SMB and *S. oralis* 89a for the ability to interfere with biofilm of typical upper respiratory tract pathogens.

**Methods:**

To investigate if soluble substances secreted by the two streptococci could inhibit biofilm development of the selected pathogenic strains, co-cultures were performed with the use of transwell inserts. Mixed-species biofilms were also produced, in order to evaluate if the inhibition of biofilm formation might require direct contact. Biofilm production was investigated by means of a spectrophotometric assay and by confocal laser scanning microscopy.

**Results:**

We observed that *S. salivarius* 24SMB and *S. oralis* 89a are able to inhibit the biofilm formation capacity of selected pathogens and even to disperse their pre-formed biofilms. Diffusible molecules secreted by the two streptococci and lowered pH of the medium revealed to be implied in the mechanisms of anti-biofilm activity.

**Conclusions:**

*S. salivarius* 24SMB and *S. oralis* 89a possess desirable characteristics as probiotic for the treatment and prevention of infections of the upper airways. However, the nature of the inhibition appear to be multifactorial and additional studies are required to get further insights.

**Electronic supplementary material:**

The online version of this article (10.1186/s12879-018-3576-9) contains supplementary material, which is available to authorized users.

## Background

Despite the presence of mechanical barriers and host immune defenses, the upper respiratory tract offers an easy access to pathogens involved in acute and chronic infections of ears, paranasal sinuses, nose and throat. The majority of upper respiratory tract infections (URTIs) are commonly mild and caused by viruses; however, URTIs can also be mediated by bacteria, representing a clinical challenge related to a higher morbidity and a chronic progress of the disease [1]. Moreover, URTIs have been associated with the presence of microbial biofilm, which results in chronic infections characterized by remitting course and resistance to medical management [2, 3]. Biofilm is defined as a “structured community of microorganisms enveloped in a self-produced polymeric matrix, adherent to an inert or living surface” [4]. Once the biofilm is established, the infection becomes more and more difficult to eradicate, because the microbes residing into the matrix are protected from host immune system and antibiotics [5]. Consequently, the microbial biofilm makes infections persistent and more refractory to treatments.

Biofilms usually consist of mixed microbial communities able to interact with other species in the surroundings and to communicate through signaling molecules [6]. These interspecies interactions may result in either mutualistic or antagonistic effects [7, 8]. The importance of the normal microbiota in the protection against URTIs has been widely demonstrated. However, an imbalance in the physiological flora composition may lead to the colonization and infection of the mucosae by opportunistic pathogens.

For instance, it has been noted that otitis-prone children were characterized by a significantly lower number of α-hemolytic streptococci in their nasopharyngeal flora than non-otitis-prone ones [9, 10], opening the possibility to administer living microorganisms as probiotics to confer health benefits to the host [11]. Indeed, the use of bacterial species deriving from healthy human oral microbiota as a probiotic for the treatment of URTIs has been proposed as a valid alternative to antibiotics, contributing to the re-establishment of a balanced flora while reducing or preventing the adhesion and colonization of potential pathogens.

Alpha-hemolytic streptococci (i.e. *Streptococcus salivarius* and *Streptococcus oralis*) isolated from human pharynx are known to be early colonizers of upper respiratory mucosae and their numeric predominance is suggestive of a healthy flora [12, 13]. Furthermore, these species possess desirable characteristics, such as production of bacteriocin like Colicin V and bacteriocin-like peptides [14, 15] and both act as pioneer colonizer. Nonetheless*, S. salivarius* and *S. oralis* own a high affinity to human mucosae, protecting epithelial cells from pathogen adherence, internalization, and potential cytotoxic effects. For this reason, these α-hemolytic streptococci represent the predominant species in the upper respiratory healthy flora and they can selectively influence the composition of the microbiota [16–19]. In the past decades, several studies have demonstrated the intranasal administration of *S. salivarius* and *S. oralis* as safe and well-tolerated strategy to reduce the risk of new episodes of acute otitis media in otitis-prone children and to decrease middle ear fluids amount in children with secretory otitis media [19–23].

In this study, we tested the hypothesis that *S. salivarius* 24SMB and *S. oralis* 89a, both isolated from a commercial product (Rinogermina®, DMG Italia Srl, Pomezia, Italy), are able to interfere with biofilm formation in vitro and to eradicate pre-formed biofilm of typical upper respiratory tract pathogens, such as *Streptococcus pyogenes*, *Streptococcus pneumoniae*, *Moraxella catarrhalis*, *Staphylococcus aureus*, *Staphylococcus epidermidis* and *Propionibacterium acnes*. In addition, the mechanisms underlying the anti-biofilm activity of the probiotic streptococci were speculated.

## Methods

### Bacterial strains and culture media

Clinically relevant upper respiratory tract pathogens were isolated from patients with URTIs at the Laboratory of Clinical Chemistry and Microbiology of IRCCS Galeazzi Institute, where they were routinely collected and stored. In particular, biofilm-producing strains of *S. aureus*, *S. epidermidis*, *S. pyogenes*, *S. pneumoniae*, *M. catarrhalis* and *P. acnes* were selected. The identification of the isolates was carried out by means of the Vitek2 Compact (BioMerieux, Marcy L’Etoile, France) and further confirmed by pyrosequencing (PSQ96RA, Diatech, Jesi, Italy), as described elsewhere [24]. Biofilm production was assessed by means of the spectrophotometric assay described by Christensen et al. [25]. *S. salivarius* 24SMB and *S. oralis* 89a were isolated from the manufactured product and identified by biochemical assays and pyrosequencing, as described above. All strains were stored at − 80 °C in proper broths enriched with 10% glycerol (VWR Chemicals, Leuven, Belgium) until testing. Brain Heart Infusion broth (BHI, bioMérieux, Marci L’Etoile, France) was used for the culture of staphylococci, BHI plus 5% of defibrinated blood (Liofilchem, Roseto degli Abruzzi, Italy) for streptococci and *M. catarrhalis*, and thioglycollate broth (TH, Oxoid, Rodano, Italy) for *P. acnes*. When performing transwell and mixed species experiments (see below), *S. oralis* and *S. salivarius* were grown in the same medium needed for the tested pathogenic strain. Before the beginning of the study, the biofilm formation ability of *S. salivarius* 24SMB and *S. oralis* 89a was assessed in each of the above-mentioned culture media, finding no significant differences among them (data not shown).

### Interaction between *S. salivarius* 24SMB and *S. oralis* 89a

To evaluate whether the reciprocal interactions between *S. salivarius* 24SMB and *S. oralis* 89a could inhibit their biofilm production, co-cultures were performed with the aid of transwell inserts (microporous PET membranes with pore diameter of 0.4 μm, 1.6 × 10^6^ pores/cm^2^) designed for 24-well plates (Falcon®, Corning, New York, NY, USA), as described elsewhere [26]. Wells were inoculated with 800 μL of *S. salivarius* 24SMB suspension and 200 μL of *S. oralis* 89a suspension were dispensed in the upper compartment of transwell inserts, and vice versa. Thereafter, mixed dual-species biofilms were also produced by inoculating 1 mL of a mixture of the two probiotic strains. In all the aforementioned experimental settings*, S. salivarius* and *S. oralis* were cultured in a 98:2 ratio (about 1.5 × 10^7^ CFU/mL of *S. salivarius* and 3 × 10^5^ CFU/mL of *S. oralis*), as that of the manufactured product. Mono-species biofilms of each strain were produced as positive controls, while medium alone was used as negative control. The experiment was performed in triplicate. Strains were incubated at 37 °C in proper conditions, and after 72 h the biofilm formation was evaluated.

### Interference on biofilm formation

To investigate if soluble substances secreted by the probiotic bacteria *S. salivarius* 24SMB and *S. oralis* 89a could inhibit biofilm formation by the tested strains, co-cultures were performed with the use of transwell inserts, as described above. Wells were inoculated with 800 μL of the target bacterial species (1.5 × 10^7^ CFU/mL) and 200 μL of a mixture of *S. salivarius* and *S. oralis* in a 98:2 ratio were inoculated in the transwell inserts (about 1.5 × 10^7^ CFU/mL of *S. salivarius* and 3 × 10^5^ CFU/mL of *S. oralis*). Thereafter, mixed species biofilms were also produced in order to evaluate if the biofilm formation by the selected pathogens requires a direct contact with the two probiotic strains to be inhibited. Mono-species biofilms of each strain were used as positive controls, while medium alone was used as negative control. The experiment was performed in triplicate for each strain. Plates were incubated at 37 °C in proper conditions. After 24, 48 and 72 h, transwell inserts and liquid medium were removed, and the amount of biofilm was evaluated by a spectrophotometric assay as described below.

### Interference on pre-formed biofilm

To investigate whether the probiotic strains were able to break an existing biofilm down, biofilm of each target strain was grown for 72 h in proper conditions and then incubated in the presence of *S. salivarius* and *S. oralis* for an additional time (24, 48 and 72 h), as previously described. Mono-species biofilm of each strain was produced as positive control, while medium alone was used as negative control. The experiment was performed in triplicate for each tested strain. At each time point, the amount of biofilm was evaluated spectrophotometrically as described below, and expressed as percentage in respect to pre-treatment level.

### Spectrophotometric assay

The amount of biofilm was quantified by means of the spectrophotometric assay developed by Christensen et al. [25], adjusting the volume of reagents for 24-well plates. Briefly, at the end of each incubation time, the culture medium was removed, and two washes with sterile saline were performed in order to remove non-adherent bacteria. After air-drying, each well was stained with 1 mL of 1% crystal violet solution (Merck, Darmstadt, Germany) for 10 min and the dye excess removed with three washes of sterile saline. Once dried, 1 mL of absolute ethanol was added to each well to solubilize the dye attached to the biofilm. An aliquot of the solubilized dye was finally transferred into a 96-wells plate for spectrophotometric reading which was performed at a wavelength of 595 nm using a microplate reader (Multiskan FC; Thermo Scientific, Milan, Italy).

### Confocal laser scanning microscopy assay

The inhibition of biofilm formation was evaluated by confocal laser scanning microscopy (CLSM) assay. Specifically, biofilms were cultured on uncoated 10 mm diameter glass microscope coverslip (VWR International Srl, Milano, Italy) in 24-well plates for 72 h using the same set-up described for transwell experiments. After 72 h of incubation at proper conditions, biofilms were gently washed with sterile saline and stained with Filmtracer™ LIVE/DEAD™ Biofilm Viability Kit (Thermo Fisher Diagnostics SpA, Rodano, Italy), according to manufacturer’s instructions. Briefly, the staining solution was prepared by adding 3 μL of SYTO9 and 3 μL of propidium iodide to 1 mL of filter-sterilized water. Biofilm samples were stained by incubation with 20 μL of staining solution for 15 min at room temperature in the dark. After incubation, samples were washed with sterile saline and examined with an upright TCS SP8 (Leica Microsystems CMS GmbH, Mannheim, Germany) using a 20× dry objective (HC PL FLUOTAR 20×/0.50 DRY) plus a 2× electronic zoom. A 488 nm laser line was used to excite SYTO9, while a 552 nm laser line was used to excite propidium iodide. Sequential optical sections of 1.27 μm were collected along the z-axis over the complete thickness of the sample. Images from at least three randomly selected areas were acquired for each coverslip. The obtained images were processed with Las X (Leica Microsystems CMS GmbH, Mannheim, Germany) and analyzed with Fiji software (Fiji, ImageJ, Wayne Rasband National Institutes of Health). The following parameters were evaluated: a) the overall volume to provide an estimation of the total biomass of the biofilm; b) the live/dead cells ratio; c) the substratum coverage, as the percentage of substrate area occupied by the biofilm.

### Cell-free extract interference on biofilm formation

To investigate the nature of the inhibition of pathogens biofilm formation by *S. salivarius* 24SMB and *S. oralis* 89a, a cell-free extract (CFE) of the supernatant was obtained. Briefly, as described above, the probiotic strains were cultured in the upper compartment of the transwell, concomitantly with each of the tested bacterial species. After 24 h of incubation (48 h for *P. acnes*), the medium from both the well and the upper chamber of the transwell was collected in a 15 ml tube (Falcon®, Corning, New York, NY, USA), centrifuged for 10 min at 4200 rpm and filtered through a 0.2 μm filter (ClearLine®, Biosigma S.r.l., Cona, Italy). The resulting CFE was divided in three vials: one was not treated to assess the effect of CFE on bacterial biofilm formation; the second was neutralized to a pH value of 7.0 using NaOH 1 M; the third was heated at 100 °C for 10 min to assess the contribution of thermolabile molecules to biofilm inhibition.

To determine the activity of CFEs, biofilm formation assay was carried out on 96-well polystyrene plates (Biosigma S.r.l., Cona, Italy) by growing the pathogenic species in fresh BHI alone (for positive control) or adding ½ (*v*/v) or ¼ (v/v) of both treated and untreated CFE. Production of biofilm was then measured by spectrophotometric assay as described above.

### Statistical analysis

Results were expressed as mean ± standard deviation and analysed for statistical significance with PRISM5 software (GraphPad, San Diego, CA, USA) using unpaired t test for CLSM assays, one-way analysis of variance (ANOVA) for the interaction between *S. salivarius* and *S. oralis* and the cell-free extract tests, two-way ANOVA the spectrophotometric assays. One-way ANOVA and two-way ANOVA were followed by Bonferroni post hoc correction. A *P*-value ≤0.05 was used as the significance level.

## Results

### Interaction between *S. salivarius* 24SMB and *S. oralis* 89a

**Fig. 1:**
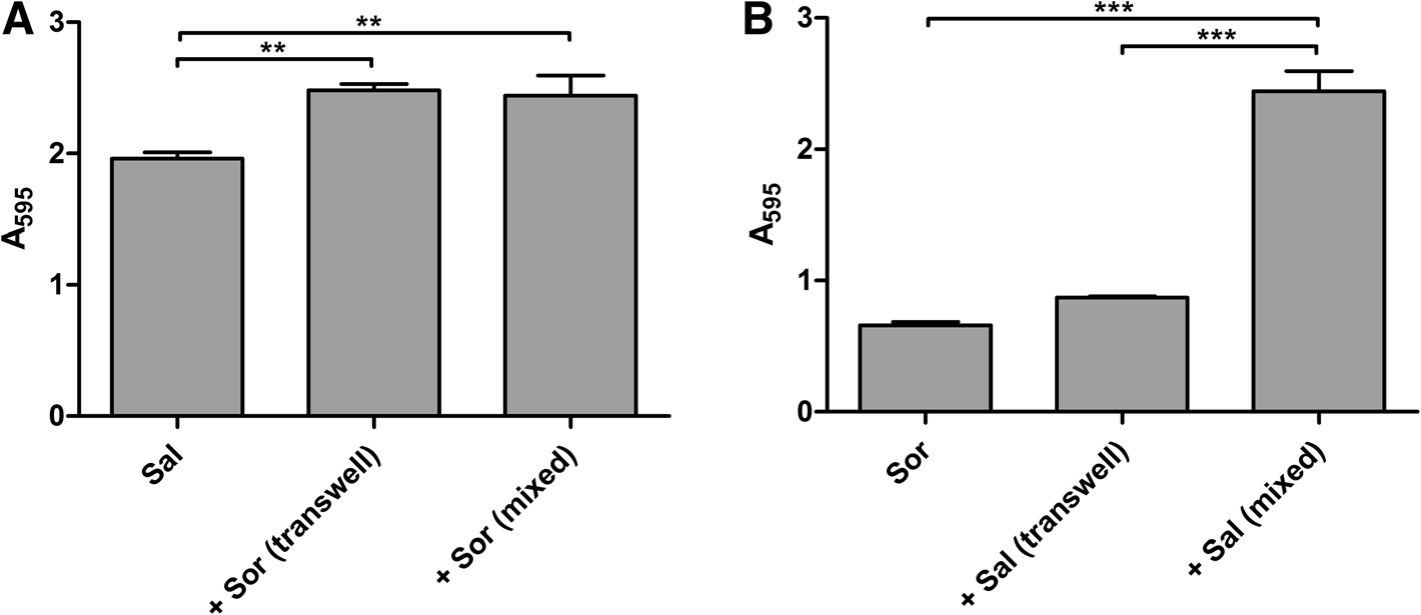
Interaction between S. salivarius 24SMB and S. oralis 89a. Data are expressed as mean absorbance ± standard deviation (*n* = 3). Sal = S. salivarius; Sor = S. oralis; ** *P* ≤ 0.01; *** *P* ≤ 0.001. Panel A shows the effect of S. oralis 89a on S. salivarius 24SMB biofilm in indirect (transwell) and direct (mixed) contact. Panel B shows the effect of S. salivarius 24SMB on S. oralis 89a biofilm in indirect (transwell) and direct (mixed) contact

The reciprocal interaction between the two probiotic strains led to an increase in biofilm production of both *S. salivarius* (21%) and *S. oralis* (24%), compared to biofilm produced when cultured separately (Fig. 1a and b). When the two species were put in direct contact (mixed) in dual-species biofilms, a significant increase of the biofilm formation was also observed, although the contribution of each strain could not be discriminated.

### Interference on biofilm formation

**Fig. 2:**
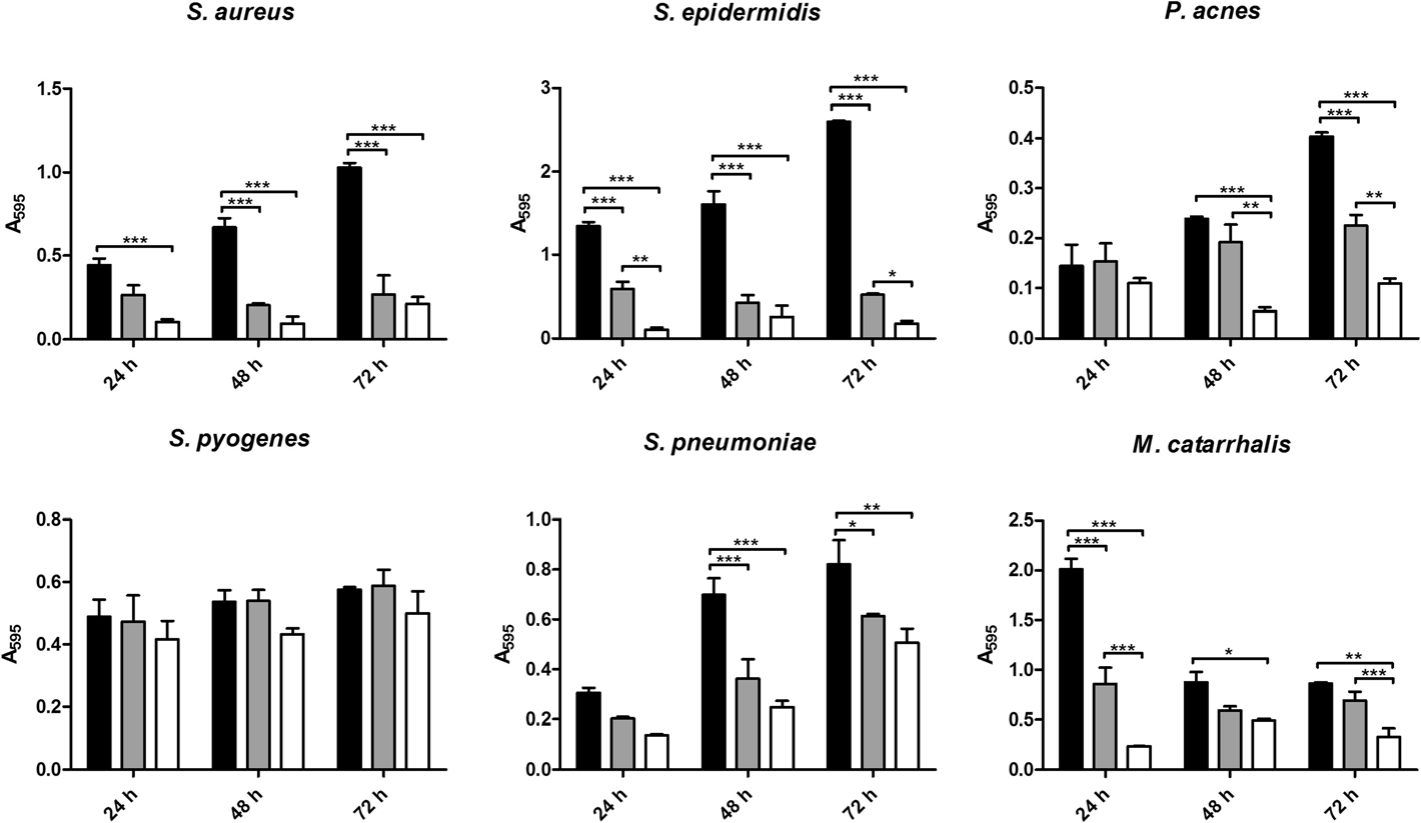
Inhibition of biofilm formation during time. Data are expressed as mean absorbance ± standard deviation (n = 3). Black bars = controls; grey bars = mixed co-cultures; white bars = transwell co-cultures; * *P* ≤ 0.05; ** P ≤ 0.01; *** P ≤ 0.001

Generally, the mixture of *S. salivarius* and *S. oralis* displayed an inhibitory activity against biofilm development of all tested bacteria, except for *S. pyogenes* whose biofilm formation was not significantly influenced by presence of the probiotic strains (Fig. 2). Biofilm production by staphylococci was strongly affected by *S. salivarius* and *S. oralis*: significant reductions were observed at all time points in both transwell (76–86% for *S. aureus* and 84–92% for *S. epidermidis*) and mixed biofilms (40–74% for *S. aureus* and 56–80% for *S. epidermidis*), compared to controls. Significant biofilm reductions were observed for *S. pneumoniae* (38–66%) and *P. acnes* (73–77%) after 48 h and 72 h of incubation in transwell experiments, while mixed co-cultures significantly inhibited *S. pneumoniae* biofilm production at 48 and 72 h (25–48%) and *P. acnes* at 72 h (44%). Finally, *S. salivarius* and *S. oralis* showed a significant inhibitory activity against *M. catarrhalis* at all time points in transwell experiments (44–88%) and at 24 h in mixed ones (57%), despite a sudden biofilm reduction in controls after 24 h might have masked the true effect of the two probiotic strains. In general, biofilm reduction was always higher in transwell experiments than in mixed ones, although such difference was not always statistically significant (Fig. 1).

### Interference on pre-formed biofilm

**Fig. 3:**
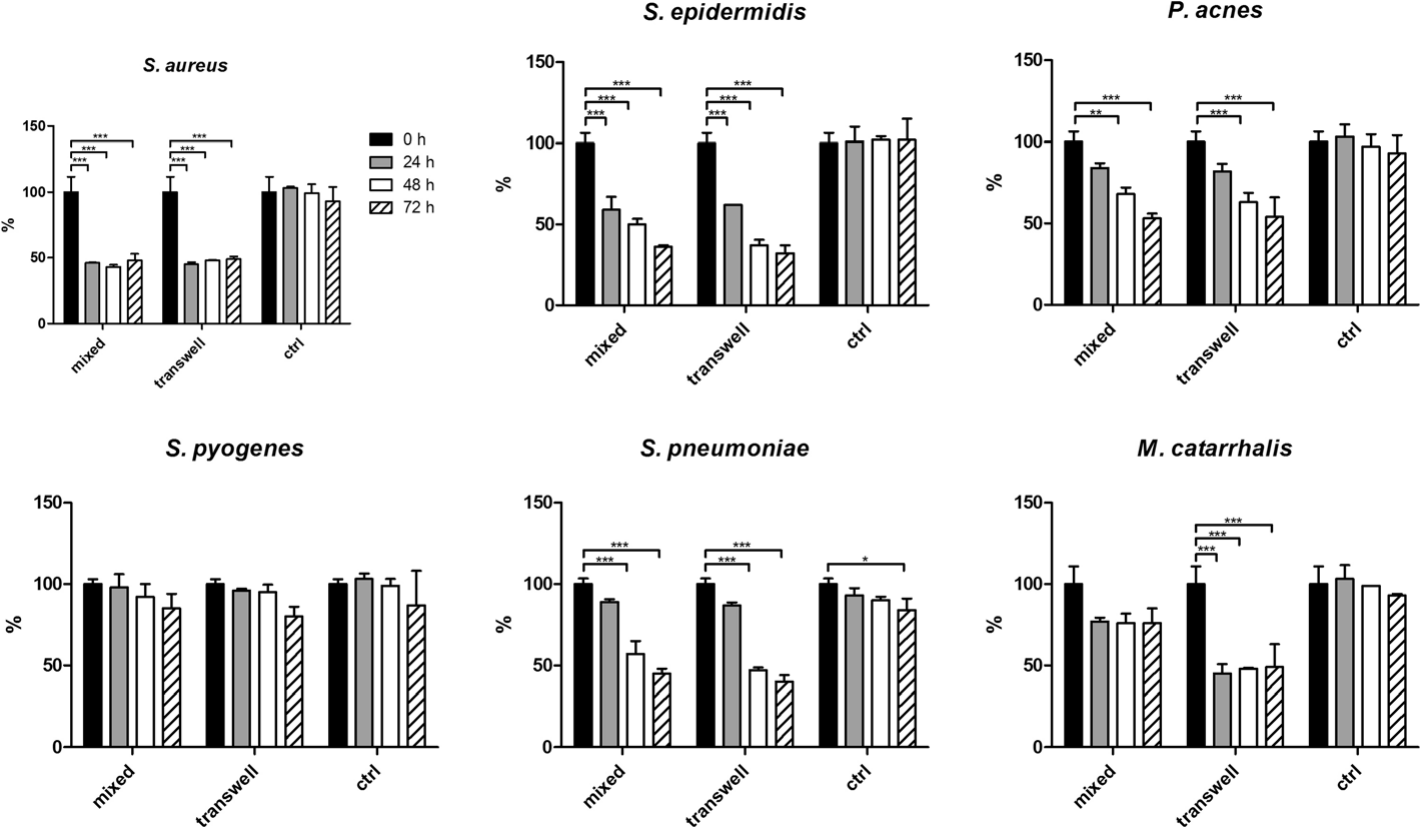
Inhibition of pre-formed biofilm during time. Data are expressed as mean percentage in respect to the pre-treatment level ± standard deviation (n = 3). Black bars = pre-treatment level; grey bars = 24 h; white bars = 48 h; dashed bars = 72 h; ctrl = untreated; * P ≤ 0.05; ** P ≤ 0.01; *** P ≤ 0.001

The combination of *S. salivarius* and *S. oralis* was able to significantly disrupt the pre-formed biofilm of all tested bacteria. Conversely, *S. pyogenes* biofilm was only slightly affected by the probiotic strains (Fig. 3). After 24 h of incubation, almost the 50% of the mature biofilm of *S. aureus* was eradicated because of the presence of *S. salivarius* and *S. oralis* in both transwell and mixed experiments, and such inhibition was maintained during time. Similarly, *M. catarrhalis* biofilm was significantly reduced in experiments using the transwell system, with a constant reduction of about 50% at all the time points. On the other hand, the anti-biofilm activity of the probiotic strains against *S. epidermidis*, *P. acnes* and *S. pneumoniae* seemed to increase during time, showing the highest percentages of biofilm reduction at 72 h (64 and 68% for *S. epidermidis*, 46 and 47% for *P. acnes* and 55 and 60% for *S. pneumoniae* in transwell or mixed co-cultures, respectively).

### CLSM assay

**Fig. 4:**
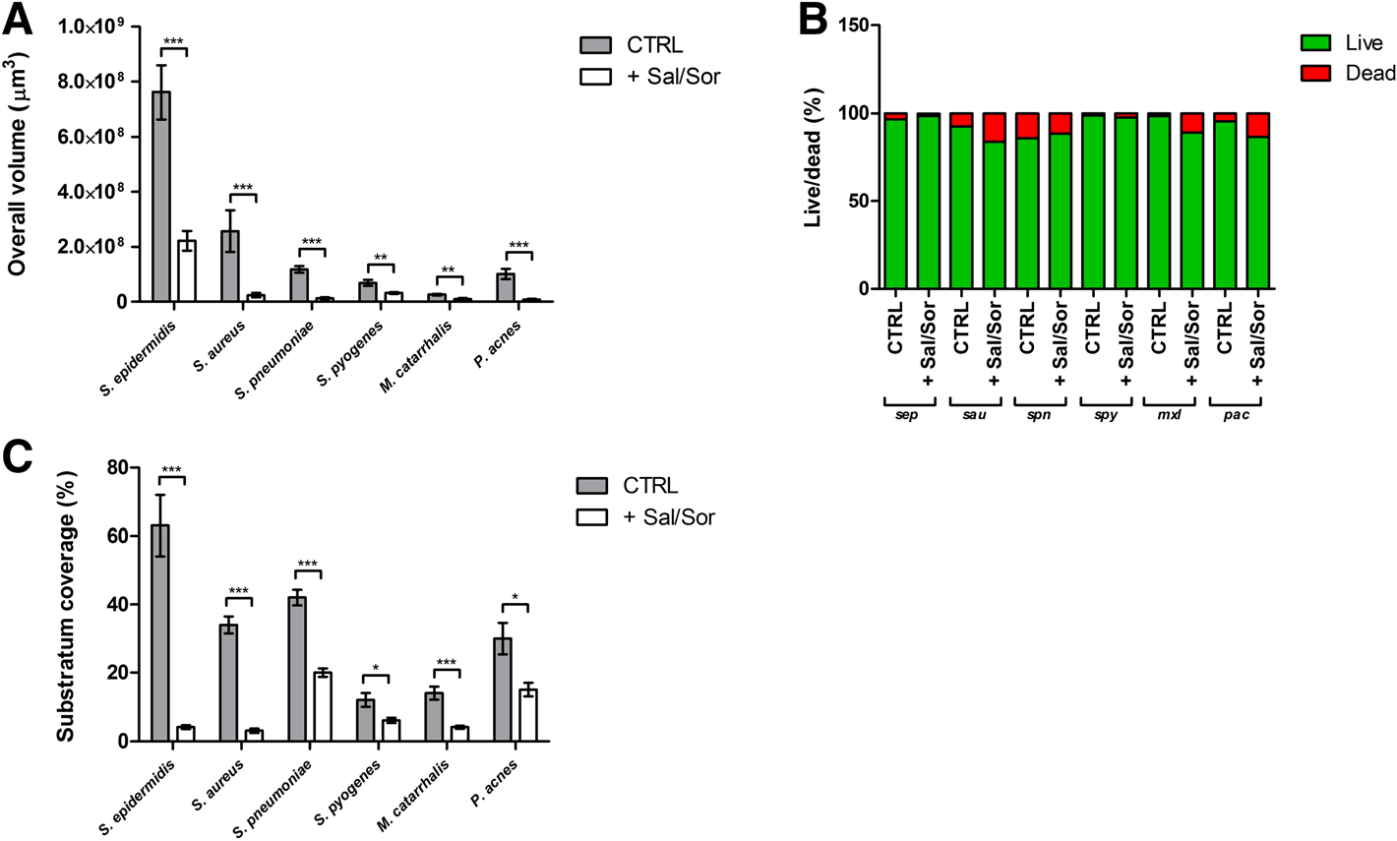
Results of confocal scanning microscopy analysis. Data are expressed as mean ± standard deviation (n = 3). CTRL = untreated samples; + Sal/Sor = samples incubated with a mixture of *S. salivarius* and *S. oralis* by means of transwell inserts; sep = *S. epidermidis*; sau = *S. aureus*; spn = *S. pneumoniae*; spy = *S. pyogenes*; mxl = *M. catarrhalis*; pac = *P. acnes*; * P ≤ 0.05; ** P ≤ 0.01; *** P ≤ 0.001

**Fig. 5:**
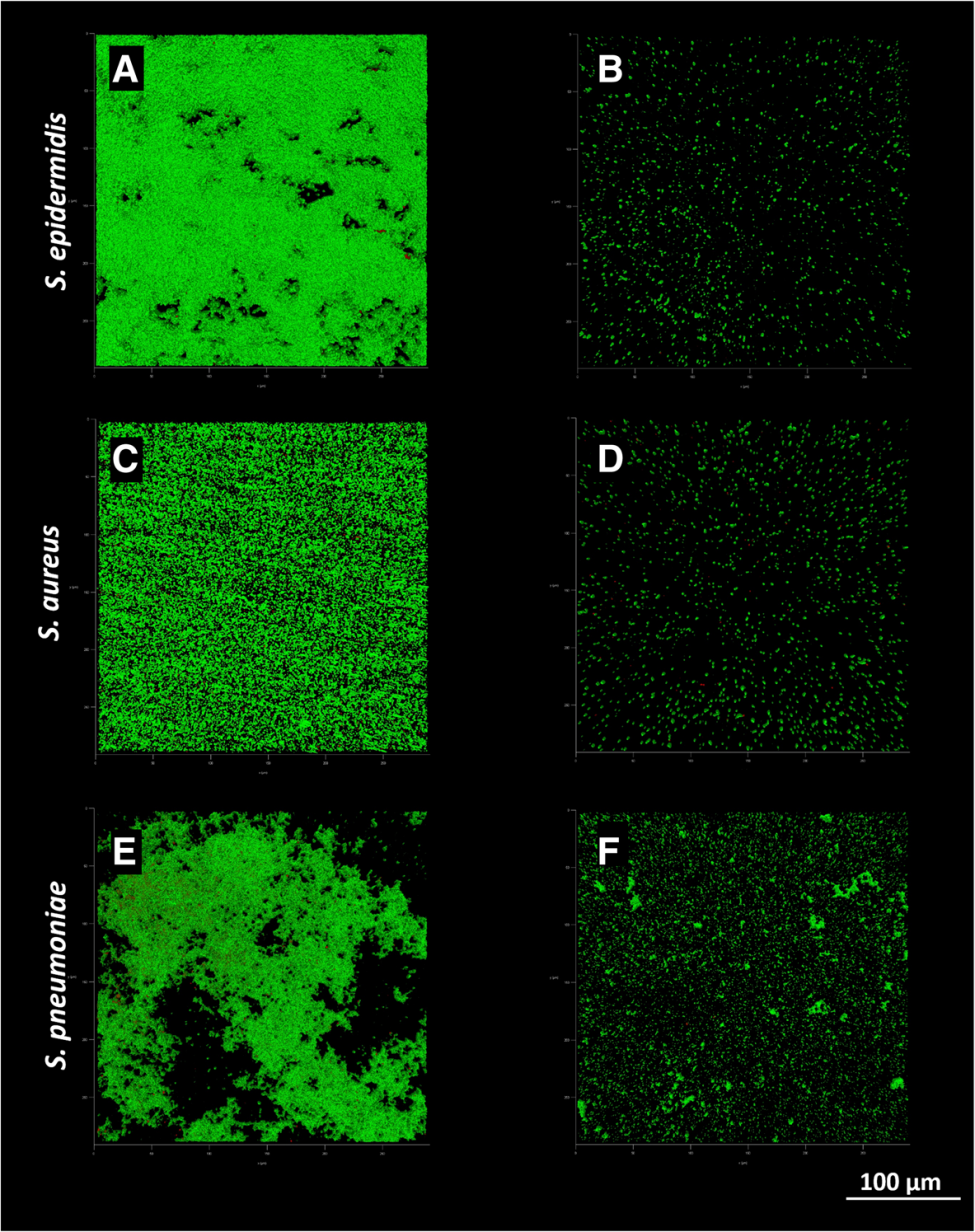
Representative images of *S. epidermidis*, *S. aureus* and *S. pneumoniae* biofilms obtained by CSLM. Panels A, C and E show control biofilms, while panels B, D and F show biofilms co-cultured in presence of the probiotic strains by means of transwell inserts. Green = live cells; red = dead cells; 40× magnification

**Fig. 6:**
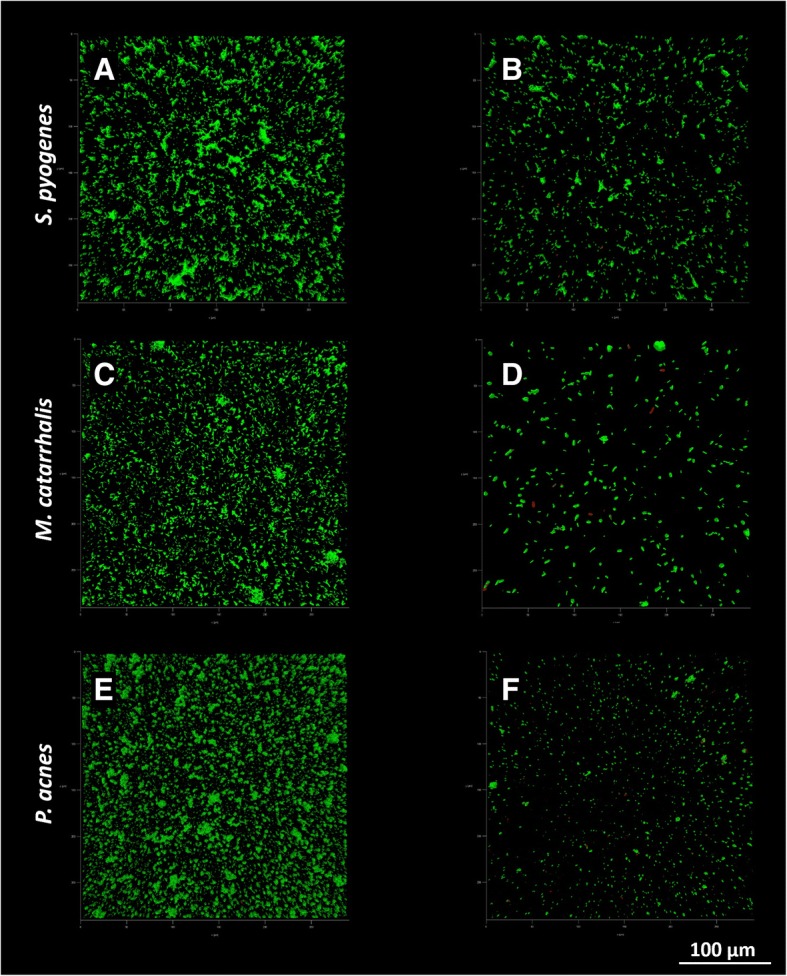
Representative images of *S. pyogenes*, *M. catarrhalis* and *P. acnes* biofilms obtained by CLSM. Panels A, C and E show control biofilms, while panels B, D and F show biofilms co-cultured in presence of the probiotic strains by means of transwell inserts. Green = live cells; red = dead cells; 40× magnification

The total biomass volume of samples incubated with *S. oralis* and *S. salivarius* in transwell experiments was significantly lower compared to control samples for all the tested strains (Fig. 4a). No differences in the live and dead cells ratio were found whit respect to treated and untreated biofilms (Fig. 4b). Substratum coverage was significantly lower for samples incubated with the probiotic strains than for control samples (Fig. 4c). As documented by CLSM images (Figs. 5 and 6), biofilms grown in presence of *S. oralis* and *S. salivarius* looked more scattered than control biofilms, especially those of *S. epidermidis* and *S. pneumoniae*.

### CFE influence on biofilm formation

**Fig. 7:**
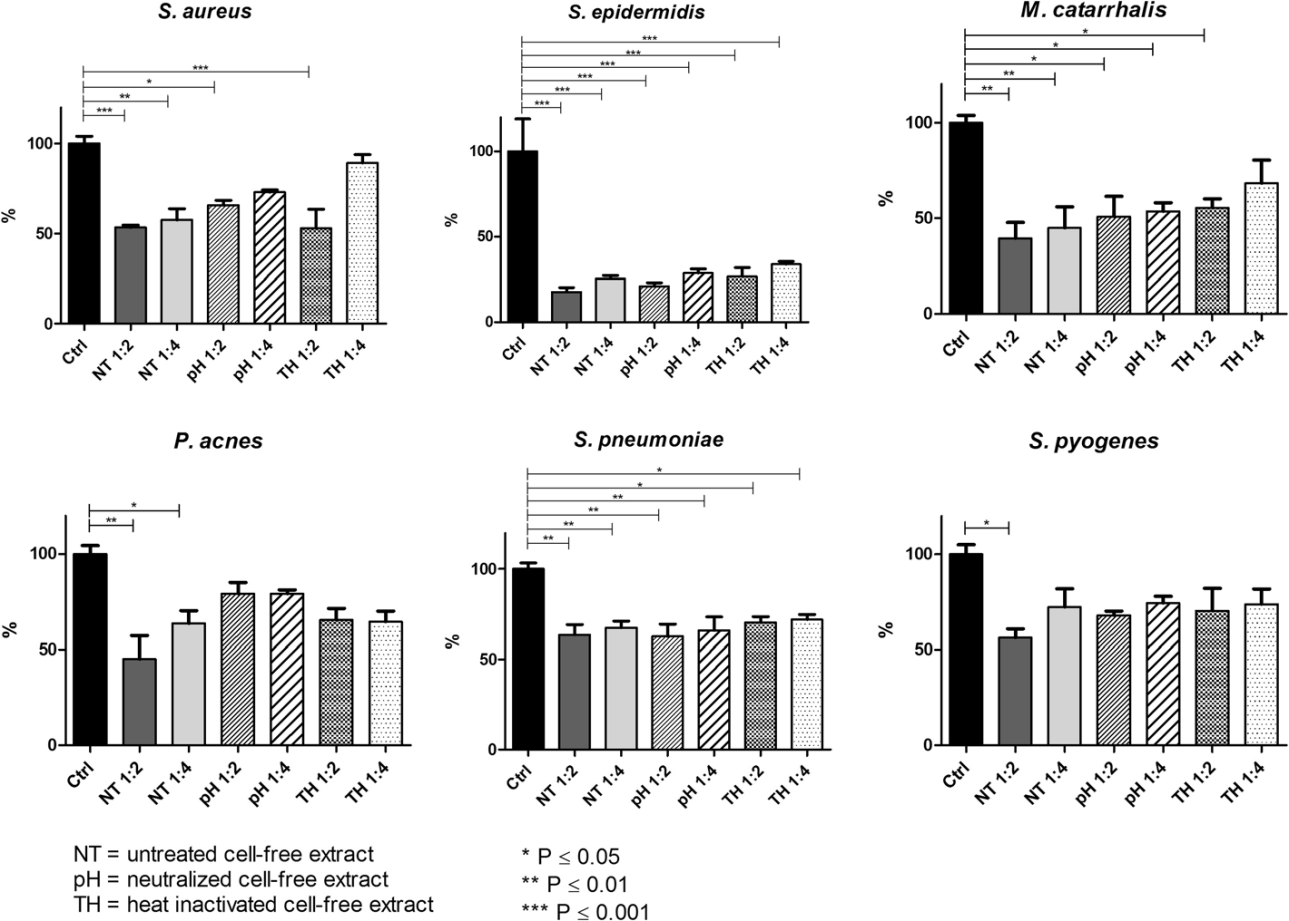
Inhibition of biofilm formation by cell-free extracts. Data are expressed as mean percentage in respect to biofilm growth control in fresh BHI broth. Black bars = control; grey tone bars = untreated cell-free extract (NT); slanted lines bars = pH neutralized cell-free extract (pH); dotted bars = heat inactivated cell-free extract (TH). * P ≤ 0.05; ** P ≤ 0.01; *** P ≤ 0.001

CFE had a significant inhibitory effect on all the tested strains with respect to the control biofilm growth, even if a general slight concentration-dependent effect was noticed (Fig. 7). All the other strains showed significant biofilm biomass reductions with all the formulations tested (45–50% and 41–51% for *S. aureus*, 49–77% for *M. catarrhalis*, 40–80% for *P. acnes*, 26–45% and 29–40% for *S. pneumoniae*, ½ and ¼ *v*/v respectively) with *S. epidermidis* being the most affected (78–86%). Differently, *S. pyogenes* was more recalcitrant to CFE addiction than the other tested species, with a significant reduction in the presence of higher amounts of CFE (CFE ½ v/v 35–49%). When CFEs were treated to neutralize the pH or heated at 100 °C to inactivate thermolabile molecules, a mild impairment of CFE activity was observed, but no significant differences were detected with respect to untreated CFEs (Fig. 7).

## Discussion

Local administration of commensal probiotics as a sort of “bacteria-therapy”, able to interfere with disease-associated disease is gaining increasing interest, finding applications in many fields [27].

Probiotics own different mechanisms that interfere with the activity of pathogenic bacteria, including: the production of antagonistic substances (e.g., bacteriocins, fatty acids, hydrogen peroxide and lactic acid), generation of environment conditions unfavorable for pathogens (e.g., competition for nutrients or pH alteration) and the competitive adhesion to human tissues preventing the colonization by harmful bacteria. Among bacterial species recently recognize as probiotic, *S. salivarius* 24SMB and *S. oralis* 89a isolated from the rhinopharynx of healthy children are suggestive strains of a healthy flora. *S. salivarius* 24SMB and *S. oralis* 89a are administered in a ratio of 98:2 by means of a nasal spray are present, with the aim of preventing ear, nose and throat diseases. The abundance of *S. salivarius* in the solution is due to its primary and predominant presence in the upper respiratory tract surfaces of humans and because its non-pathogenic behavior in healthy individuals [28]. In particular, the strain 24SMB was isolated in 2012 and selected as a promising probiotic due to the absence of virulence traits and antibiotic resistance genes and its ability to inhibit *S. pneumoniae* growth [21]. Santagati et al. demonstrated its safety and tolerability, and its capability to colonize the rhinopharynx when administrated as a nasal spray [22].

Conversely, *S. oralis* 89a was isolated from a recalcitrant healthy child during a tonsillitis outbreak and it was found to be able to inhibit the growth of group A streptococci in vitro [29]. Since then, this strain has been used in in vitro and in vivo studies to evaluate its clinical effects on streptococcal tonsillitis and otitis media [19, 20, 30–32]. The whole genome of this strain was recently sequenced, identifying the gene encoding for the bacteriocin Colicin V and for tolerance to Colicin E2 [14].

As a preliminary step, the reciprocal interaction between *S. salivarius* 24SMB and *S. oralis* 89a was assessed. Using the transwell device, the two strains were physically separated by a membrane that permitted only the passage of diffusible molecules form one compartment to the other. In both cases, a significant increase in biofilm formation was observed, suggesting a possible positive synergistic effect between the two strains. Furthermore, when *S. salivarius* 24SMB and *S. oralis* 89a were simultaneously cultured in direct contact, the resulting biofilm appeared to be like the sum of the two strains grown individually. Unfortunately, the contribution of a single strains in mixed biofilms was not evaluated, being the used staining technique not species-specific, representing a limit of the study.

Then, the anti-biofilm activity resulting from the combination of *S. salivarius* 24SMB and *S. oralis* 89a against pathogens of the upper airways was investigated. In particular, the anti-biofilm activity was tested against *S. pneumoniae*, *S. pyogenes* and *M. catarrhalis* being the most common bacterial pathogens causing acute otitis media and bacterial pharyngotonsillitis [33, 34]. Moreover, the anti-biofilm effect was assessed on *S. aureus*, involved in 50% of recalcitrant chronic rhinosinusitis [35], and coagulase negative staphylococci and anaerobes, including *P. acnes* [36].

Biofilms are comprised of microorganisms enclosed in a hydrated self- produced polymeric matrix attached to a solid surface. They represent an important cause of chronic infectious diseases of the upper airways, including recurrent middle ear diseases, chronic rhinosinusitis and recurrent pharyngotonsillitis [2], but also teeth or in implant-associated infections. Biofilm-related infections are often resistant to antibiotic therapy, posing serious concerns about the infection control. Frequent antibiotic intakes may have deleterious effects due to the depletion of the commensal microbiome and the subsequent colonization by microorganisms that are less susceptible to the prescribed antibiotics [37]. In this context, the use of probiotics able to disperse pathogens biofilm may be therapeutically beneficial and may offer a valid alternative or a coadjuvant treatment to conventional antimicrobials.

With the aim to test the aforementioned hypothesis, in the present study, co-culture experiments by means of the direct or indirect culture of the probiotic strains with the respiratory tract pathogens was performed. The combination of probiotics was able to both inhibit the biofilm development and to disperse the already established biofilms of all the tested pathogens with the exception of *S. pyogenes*. This behavior is not surprising; the inhibitory activity of *S. salivarius* 24SMB against *S. pyogenes* depends on the choice of the growth media [21]. Indeed, Santagati and co-workers described how *S. salivarius* was not able to inhibit *S. pyogenes* in Todd Hewitt broth supplemented with blood, while an increase in the inhibitory activity was observed on Columbia blood agar. In our experimental setting, the lack of activity against *S. pyogenes* could also be explained by the lack of supplementation of yeast extract, glucose or calcium salts in the growth medium, which are necessary supplement for an optimal production of bacteriocins [38, 39].

A more detailed investigation was carried out by CLSM analysis, which considered additional outcome variables as live/dead cells ratio and percentage of substratum coverage. Specifically, the total biomass volume was significantly lower when the tested pathogens were incubated with probiotics compared to that of controls. Differently to what observed in the spectrophotometric assay, the higher sensitivity of the CLSM analysis allowed to appreciate a significant biomass reduction also for *S. pyogenes*. Indeed, while the spectrophotometric assay allows the semi-quantitative measurement of the air-dried biofilm biomass, CLSM gives the possibility to collect three-dimensional images of hydrated biological structures without fixation [40]. This non-destructive technique has radically transformed optical imaging in biology and microbiology, providing a useful tool for the examination of the structure of biofilms.

Concerning the live to dead cells ratio, no differences between treated and untreated samples were found, indicating a prevalent inhibitory effect rather than a potential bactericidal activity of the probiotic strains. On the contrary, substratum coverage was significantly lower in treated biofilms, with particular regard to that of *S. epidermidis* and *S. aureus*, which appeared more scattered than controls.

Recent studies shown that chemical interactions through secretion of molecules by different microbial species may affect spatial biofilm structure, regulating both its formation and dispersion [41–43]. Recently, Santagati et al. described the presence of a blpU-like bacteriocine cassette in the genome of *S. salivarius* 24SMB, which has been shown to mediate intra- and interspecies competition with inhibitory activity against *S. pyogenes* and *S. pneumoniae* and also to provide competitive advantage in colonization in vivo [15, 44]. However, no other genes responsible for production of bacteriocine (i.e. salivaricins, commonly produced by other *S. salivarius* strains) were identified throughout the genome [15]. Similarly, *S. oralis* 89a possess a locus for the production of a Colicin V, a proteolitically processed peptide antibiotic, which kills sensitive cells by disrupting membrane potential [45]. Interestingly, *S. oralis* 89a is characterized by the presence of *luxS* gene, responsible for the production of the quorum-sensing molecule AI-2, involved in cell-to-cell communication and able to influence the expression of virulence factors, motility and biofilm formation [46]. In particular, controlled concentrations of AI-2 are able to promote mutualistic biofilm formation and to influence structure and composition of other commensal streptococcal species biofilm [47, 48]. Unluckily, the genome sequence of *S. salivarius* 24SMB is not available in public databases and no information on the presence of quorum-sensing clusters involved in biofilm formation and regulation (e.g., Rgg transcriptional regulators family) are available.

Nonetheless, signaling is restricted only to those species with appropriate receptors, suggesting that other kind of unspecific interactions may play an important role in determining biofilm spatial structure [49]. For example, metabolic end products like lactic acid and hydrogen peroxide produced by streptococci,

can act with a broader spectrum by cause acid and oxidative stress, respectively. As expected, the presence of *S. salivarius* 24SMB and *S. oralis* 89a in the co-cultures slightly lowered the pH in all the cases except for *S. pyogenes* and *S. pneumoniae* (data shown in Additional file 1). Since the inhibitory activity was observed also for the two streptococci, it can be supposed that alteration in pH is not the only mechanism of action, but other specific interactions might occur.

Finally, a high reduction of biofilm biomass in transwell co-cultures was observed. This event might suggest that the anti-biofilm activity of the probiotic mixture is mediated by diffusible molecules secreted by the probiotic strains, rather than depending on a mechanism requiring physical contact. This was confirmed by supplementation of the cell-free extract to the medium, displaying an effect comparable to that observed in the transwell co-culture. In the attempt to elucidate the nature of the inhibition, the cell-free extracts were neutralized to a pH of 7.0, to evaluate the contribution of the acidic environment resulting from the streptococcal fermentation or were heated, to eliminate all the thermolabile secreted molecules. Even though, both the treatments did not completely impair the inhibitory effect of the cell-free extract, indicating a likely multifactorial and strain-specific strategy. Furthermore, the contribution to biofilm formation between pathogenic and probiotic strains in mixed species biofilms was not discriminated. Indeed, mixed biofilms were not analyzed by confocal microscopy, because of the lack of species-specific dyes able to differentiate the presence of different microbes. Stains able to discriminate among different bacteria should be used in future studies in order to investigate the role of probiotic strains in the biofilm production by pathogenic bacteria.

As the goal of the probiotics tested in this study is to create a barrier against pathogens, an interesting issue would be to investigate if pathogens are able to invade and establish within pre-existing probiotic biofilms.

## Conclusions

In this preliminary study, we demonstrated the capability of *S. salivarius* and *S. oralis* to interfere with the biofilm formation capacity of the upper airways pathogens and disperse their pre-formed biofilms. The nature of inhibition seems to be multifactorial, involving both specific and unspecific mechanisms. However, additional studies are required to get further insights into the mechanisms underlying these interactions at molecular level.

## Additional files

Additional file 1: Broth pH after 72 h. Culture medium pH measurement after 72 h of growth. (DOCX 12 kb)

## Abbreviations

CLSM: Confocal laser scan microscopy
URTIs: Upper respiratory tract infections
