# Supplementary material for: Probiotics Streptococcus salivarius 24SMB and Streptococcus oralis 89a interfere with biofilm formation of pathogens of the upper respiratory tract
Source: BMC Infect Dis. 2018 Dec 13;18:653. doi: 10.1186/s12879-018-3576-9 (PMC6292094; doi:10.1186/s12879-018-3576-9)
Supplement: Supplementary file 1 — Broth pH after 72 h. Culture medium pH measurement after 72 h of growth. (DOCX 12 kb) [file 12879_2018_3576_MOESM1_ESM.docx]

|  | **pH 72h** |
| --- | --- |
| **BHI** | 7 |
| **Sal/Sor** | 4,5 |
| **Sep** | 6 |
| **Sep + Sal/Sor** | 5 |
| **Sau** | 6 |
| **Sau + Sal/Sor** | 5 |
| **BHI + blood** | 7 |
| **Sal/Sor** | 5,5 |
| **Spn + Sal/Sor** | 5,5 |
| **Spy** | 5,5 |
| **Spy + Sal/Sor** | 5,5 |
| **Mxl** | 7 |
| **Mxl + Sal/Sor** | 5,5 |
| **Thi + blood** | 6,5 |
| **Sal/Sor** | 4,5 |
| **Pac** | 6 |
| **Pac + Sal/Sor** | 5 |

Additional file 1. Broth pH after 72 hours.

BHI, Brain Heart Infusion broth; Thi, Thioglycollate; Sal, *Streptococcus salivarius*; Sor, *Streptococcus oralis*; Sep, *Staphylococcus epidermidis*; Sau, *Staphylococcus aureus*; Spn, *Streptococcus pneumoniae*; Spy, *Streptococcus pyogenes*; Mxl, *Moraxella catarrhalis*; Pac, *Propionibacterium acnes*.
